# Presenting a Social Value Database and Simulator for Public Health

**DOI:** 10.1093/eurpub/ckac129.425

**Published:** 2022-10-25

**Authors:** A Cotter-Roberts, K Ashton, A Stielke, M Dyakova

**Affiliations:** WHO CC, Public Health Wales, Cardiff, UK

## Abstract

**Background:**

There is increasing recognition that Public Health Institutes need to build on the traditional value for money approach, to find ways to capture, measure and show the full range of their outcomes, impacts and related value. As part of a drive to measure value and impact in public health and demonstrate how investment in health can contribute to an Economy of Well-being, Public Health Wales has developed an interactive database to capture and illustrate the social value of public health services and interventions.

**Methods:**

Scoping reviews of both academic and grey literature were undertaken to populate a database of health economics evaluations of public health interventions, focusing on Social Return on Investment (SROI). In addition, a simulated methodology was developed which allows the evidence to be manipulated and made relevant to individual contexts to help inform investment decisions at a local level.

**Results:**

To date, the database has accumulated an excess of 50 SROI evaluations of various public health interventions, across areas including mental health, behaviour change, physical activity, nutrition, employment and primary care. The evaluations are based on European and International contexts, are published in both grey and academic sources, and are of varying quality.

**Conclusions:**

SROI is a credible method for measuring the value of wider social, economic and environment outcomes achieved from public health interventions. The Social Value Database and Simulator presents a collation of studies and analysis utilising innovative health economics methods.

**Key messages:**

• Public Health Wales’ Social Value Database and Simulator collates economic evaluations of public health interventions, to be used by policy makers to enable improved investment in health and well-being.

• Social Return on Investment is a credible method for measuring the wider impact created by public health interventions.

